# Suitability of a Programme for Improving Interprofessional Primary Care Team Meetings

**DOI:** 10.5334/ijic.4179

**Published:** 2018-12-13

**Authors:** Jerôme Jean Jacques van Dongen, Marloes Amantia van Bokhoven, Wilhelmus Nicolaas Marie Goossens, Ramon Daniëls, Trudy van der Weijden, Anna Beurskens

**Affiliations:** 1 Research Centre for Autonomy and Participation for People with Chronic Illnesses, NL; 2 Faculty of Health, Zuyd University of Applied Sciences, Heerlen, NL; 3 Department of Family Medicine, Care and Public Health Research Institute (CAPHRI), Maastricht University, Maastricht, NL

**Keywords:** interprofessional collaboration, interprofessional team meetings, integrated care, qualitative research, chronic diseases, process evaluation

## Abstract

**Introduction:**

Primary care is increasingly being confronted with complex health care demands stemming from both biomedical and psychosocial problems of people with chronic diseases. Interprofessional collaboration is needed to enhance person-centredness and coordinate care provision in an efficient manner, which should eventually result in high-quality and integrated care. In primary care, collaboration often occurs through periodic interprofessional team (IPT) meetings. We have developed a multifaceted programme (including a reflection framework, training activities and a toolbox) to enhance team functioning in terms of improved person-centredness and efficiency of meetings. The aim of this study was to evaluate the perceived suitability and potential impact of this programme. Eventually, findings of this evaluation should contribute to understanding the suitability of the programme and optimizing its design.

**Methods:**

A prospective process evaluation was conducted, using a mixed-methods approach. Six primary care IPTs participated. Data collection included observations of team meetings, semi-structured interviews with team chairpersons, a focus group meeting, and a questionnaire for all team members. Qualitative data were analysed using directed content analysis and quantitative data using descriptive statistics.

**Results:**

The results show that, on the whole, the programme was appreciated. Most progress was perceived regarding structure and organization. Chairs perceived increased awareness of person-centredness and team processes. They perceived the training activities as useful and instructive, and valued peer feedback and on-the-job coaching as the most effective strategies. Findings from the questionnaire showed a tendency in the desired direction for all variables.

**Conclusion:**

To conclude, the programme can be considered as a suitable approach for improving team functioning. However, enhancing person-centredness requires additional training/practice and on-the-job coaching. Lastly, the programme should be context-specific, flexible in use, and preferably delivered and mediated by an external facilitator at the workplace.

## Background

Primary health care is being confronted with an increasing number of people with multiple chronic diseases, requiring complex health and social care services. Integrated care systems appear to offer an ideal platform for optimising patient-centred care in this demanding environment [1]. A key element in delivering high-quality and integrated care is interprofessional collaboration (IPC) [2,3]. In their review, Zwarenstein and colleagues describe IPC as a process in which different professional groups work together to positively impact on health care [4]. IPC is perceived as an ongoing interpersonal process of shared goal setting and decision making, which occurs among professionals from a diversity of disciplines, and a patient system to optimize the management of chronic disease [5]. In the Dutch primary care setting, IPC often takes place through periodic interprofessional team (IPT) meetings. These meetings vary in terms of setting, duration, frequency, number of participants, disciplines and numbers of patients discussed. By way of illustration, an average team may comprise family physicians, practice nurses, occupational therapists, physical therapists and district nurses [6]. Based on a needs assessment encompassing various qualitative studies [6,7,8,9] and a scoping review [10], we concluded that there is room to improve the functioning of these IPT meetings. Often, meetings appeared to be unstructured and lacked clear coordination and leadership, and therefore seemed to lack efficiency. Moreover, observations showed that person-centredness was often lacking in team meetings [6]. Findings also emphasized the essential role of the teams’ chairpersons, who, in addition to structuring the meetings, should also act as change agents in guiding team development over time [11].

Practice-based IPC interventions appear to be able to improve healthcare processes and outcomes [4,12]. In addition, there is evidence that teamwork innovations can promote better communication, better relationships and greater satisfaction among the workforce [13]. Other studies have also demonstrated that multifaceted intervention programmes, tailored to the needs of the teams, are most effective [14]. Despite the lack of high-quality evidence, some studies show that interventions including training activities have positive effects on the effectiveness of interprofessional teams [12,15]. With this knowledge, we systematically (by means of action research) developed a multifaceted programme to improve the functioning of IPT meetings [11]. The programme comprises aspects of all three main types of interprofessional interventions as described in Reeves and colleagues’ interprofessional framework: interprofessional education; interprofessional practice; and interprofessional organization [16]. The multifaceted programme primarily focuses on organizing and structuring IPT meetings, enhancing person-centredness and guiding team development over time. Findings from the action research study confirm the important role of the chairperson being a change agent who guides team reflection and development. To be effective, the programme should also be customizable and tailored to the teams’ specific contexts and dynamics [11].

In order to contribute to understanding the way the programme is implemented in the complex primary care setting, how it works in practice, and which factors contribute to its success or failure, we conducted a thorough process evaluation. The aim of the process evaluation reported on here was to examine the suitability of the programme and optimizing its design. In addition to suitability, we examined the potential impact of the programme on team climate, efficiency and person-centredness.

## Methods

We conducted a process evaluation [17] using a mixed-methods approach, including both qualitative and quantitative data. We used principles of the Medical Research Council Process Evaluation Framework to guide the process [17], and formulated the following research questions:

1. To what extent was the programme implemented?
2. How did participants experience the programme?
3. What was the programme’s potential impact on team climate, efficiency and patient centredness?

### Setting and participants

Six interprofessional teams working in primary care were recruited by means of convenience sampling, using the researchers’ network and contacts of a regional care group (Huisartsen Oostelijk Zuid-Limburg). Teams were included if they periodically conducted IPT meetings, including three or more health care professionals from different disciplines. In each team, a chairperson and a co-chair should be willing to take part in the training activities. The team meetings, arranged by one of the team members, were part of the regular care process and not specifically initiated for this study. Team members received oral and written information about the content of the study and confidentiality of the data collection and analysis.

### Content and scope of the programme

**Figure 1:**
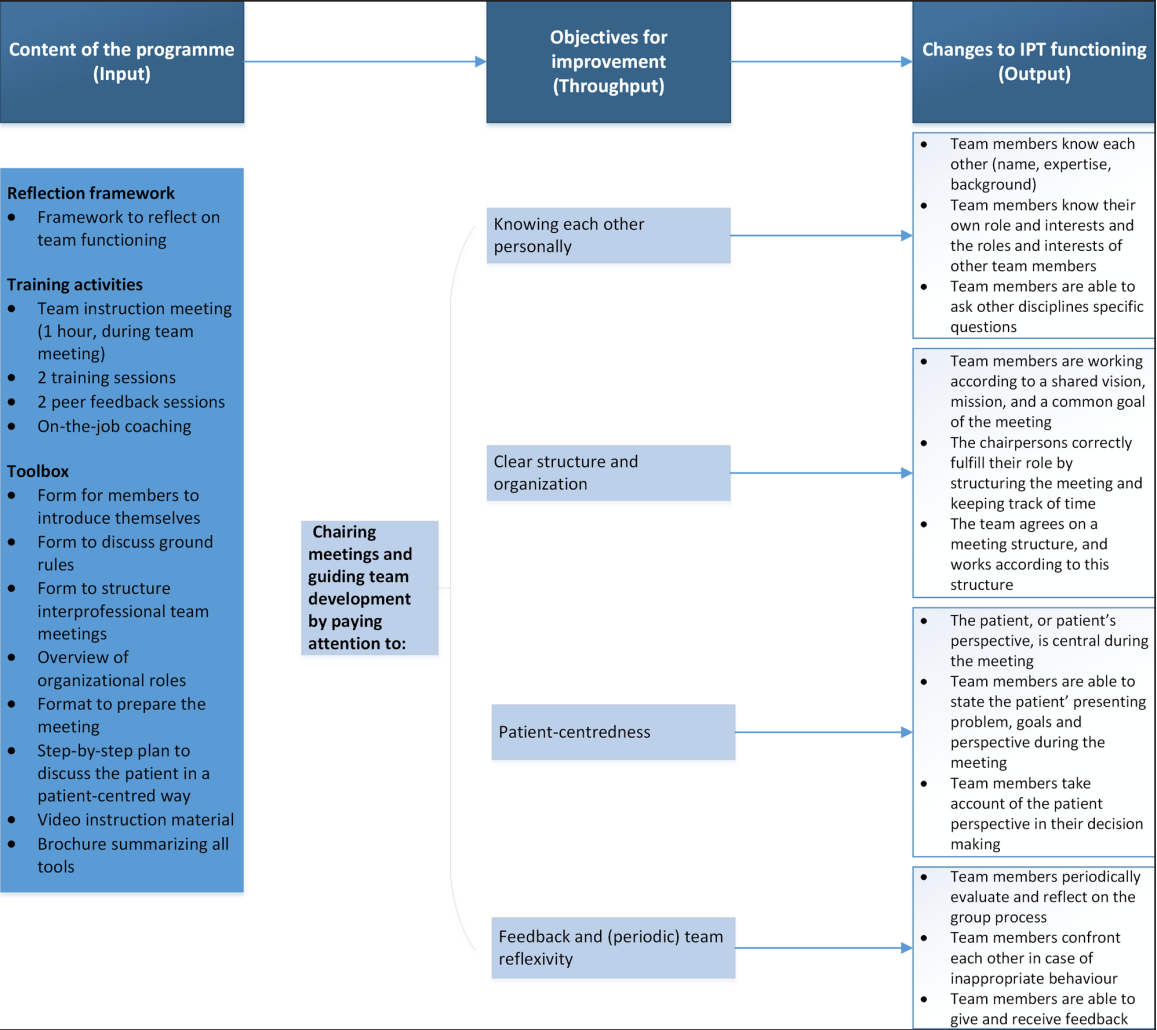
Logic model for the programme to improve IPT functioning.

The programme was developed through action research [11] and aims to improve IPT functioning by focusing on five main objectives for improvement: (1) knowing each other personally, (2) clear structure and organization, (3) person-centredness, (4) feedback and team reflexivity, and (5) chairing meetings and guiding team development. A logic model of the structure of the programme and it’s connection with the objectives for improvement is presented in Figure 1. The programme’s backbone is formed by a framework that can be used by interprofessional primary care teams to reflect on the functioning of their IPT meetings (Supplementary file 1). Team reflection should enable the programme to be customized and adapted to a team’s specific context and learning objectives. The programme also includes a team instruction meeting and multifaceted training for the chairperson and co-chair. The training course comprises two sessions, one focusing on organizing and structuring meetings, and the second primarily focusing on safeguarding person-centredness. Both sessions involve training leadership competences and use of the tools. The training course also includes two peer feedback sessions and on-the-job coaching. On-the-job coaching comprises observation of an IPT meeting and provision of feedback and feedforward immediately afterwards. In addition, a toolbox including instructive video materials and a brochure with information about the supporting tools is part of the programme. Teams are free to adjust the tools to their specific needs and apply them as appropriate. Chairpersons were asked to instruct and encourage the other team members. By focusing on the chairpersons as change agents, we intended to improve team functioning.

### Data collection

**Figure 2:**
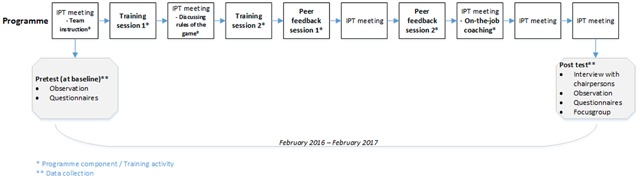
Timing of data collection.

Data were collected between February 2016 at baseline (pretest), and February 2017 (posttest) within three months after the last training activity, see Figure 2. Table 1 offers an overview of the three research questions and data collection methods. Over a one-year period, participating teams used the programme and had the opportunity to gain experience with the new approach, and to experiment with the different supplementary tools in practice. Before the observations and interviews took place, oral informed consent was obtained from all participants.

1. To what extent was the programme implemented?

**Table 1:** Research questions and data collection methods.

	Interviews with chairpersons	Observations of IPT meetings	Questionnaire	Focus group meeting (+ additional interviews)

*1. To what extent was the programme implemented?*		X		
*2. How did participants experience the programme?*	X			X
*3. What was the programme’s potential impact on team climate, efficiency and person-centredness?*			X	

#### Observations

Observations of team meetings were conducted before and after the training course to ascertain the extent to which the programme was implemented. Access to the meetings was arranged for the researchers by the teams’ chairpersons. Two researchers observed the meetings, took field notes and collected background data using an observation guide focusing on the presence and implementation of the different components of the programme (Supplementary file 2).

2. How did participants experience the programme?

#### Interviews

To explore the experiences of the participating chairpersons and co-chairs about *following/using* the programme, we conducted individual interviews. We intended to interview at least one of the two chairpersons for each participating team at posttest. The interview guide (Supplementary file 3) started with an open-ended question to discover respondents’ experiences with taking part in the programme. Other questions related to the barriers and facilitators regarding the suitability, added value and possible improvements of the programme, the chairpersons’ role and perceived personal growth. The interviews were conducted by JvD, lasted between 30 and 45 minutes, and were recorded using a voice recorder.

#### Focus group meeting

In order to explore experiences regarding the suitability and added value of the programme among team members, we organized a focus group meeting (two hours) with representatives from each of the six teams, other than the chairpersons who had taken part in the training course. By way of purposive sampling, we asked each team to delegate one or two team members to represent their teams. An experienced researcher (MvB) moderated the meeting and guided the discussion, while a second researcher (JvD) was responsible for facilitating the meeting and taking notes. The moderator guided the meeting using an interview guide based on Supplementary file 3.

3. What was the programme’s potential impact on team climate, efficiency and person-centredness?

#### Questionnaire

To examine the programme’s potential impact on team climate and person-centredness, we presented a questionnaire including 48 questions at pretest and posttest to fill out by all members attending the meeting, anonymously (Supplementary file 4). The first part of the questionnaire (Q1–38) focused on team climate. Since team climate appears to be an important characteristic of successful teams [18,19], we opted for the Dutch Team Climate Inventory (TCI), which is known to be a reliable and valid instrument [20]. The Dutch TCI contains 38 questions covering 3 domains, and has a 5-point response scale ranging from ‘strongly disagree’ to ‘strongly agree’, with higher scores indicating a better or more desirable team climate. The second part of the questionnaire (Q39–48) contained 6 questions regarding person-centredness and 4 regarding team efficiency. For these questions we used a 7-point response scale ranging from ‘strongly disagree’ to ‘strongly agree’. To assess person-centredness, we included the ‘patient involvement’ domain of the Collaborative Practice Assessment Tool (CPAT), which can be regarded as a reliable tool for assessing levels of collaborative practice [21]. Questions regarding efficiency focused on perceived team efficiency, ending with the question to rate team functioning on a scale from 1 (very bad) to 10 (very good).

### Analysis

Qualitative data were analysed by means of directed content analysis [22]. This variant of qualitative content analysis, described by Hsieh and Shannon, combines a deductive and an inductive approach. Within this approach, existing theory and research results are used to develop a coding scheme in advance, to bring more focus in analysing the data [23].

The audio-recorded interviews were transcribed verbatim. A detailed description of each observation was made, based on the focal points presented in Supplementary file 2. The summary was supplemented with field notes about significant events and non-verbal communication. Data analysis was completed by two researchers (SB and JvD). Both analysed and coded all transcripts independently and repeatedly in order to familiarize themselves with the data. They coded with the assistance of Nvivo 10 software, and compared and discussed their findings until consensus was reached [24]. The same initial coding scheme was used for both observations and interviews, based on the five main objectives for improvement. If necessary, new codes were added. In the next step, codes assigned to the interviews and observations were grouped into themes. Finally, connections between the themes were explored. Findings derived from the focus group were analysed similarly and compared with data derived from the interviews. Quantitative data from the questionnaire were analysed by descriptive statistics (frequencies, means per domain analysed, both per team and as mean overall scores) using Statistical Software package for Social Sciences (SPSS) version 23. Given the clustering of data per team, we calculated means per team.

#### Trustworthiness

Field notes and written comments were used in the analysis process to enhance the trustworthiness of the study. To increase credibility, JvD and SB analysed the data independently and discussed and compared results, consulting MvB in case of disagreement. Furthermore, combining data from both observations and interviews, known as methodological triangulation, provided additional perspectives and enhanced credibility [25]. All observations were conducted by two researchers in order to ensure independence and avoid blind spots. To increase accuracy, validity and credibility, we conducted a member check of the focus group meeting: a summary of the main findings was sent to the participants, giving them the opportunity to reflect.

## Results

The programme was implemented between February 2016 and February 2017. Data were collected until March 2017. Characteristics of the six participating teams are presented in Table 2. Table 3 offers an overview of the data collection and response rates. Regardless our selection criteria of selecting teams that already conduct IPT meetings, we also included a beginning team (team 6). This team was set up at baseline, and offered us additional perspectives. Consequently this team could not be observed at pretest. A total of five observations at pretest and six observations at posttest took place. Additionally, the chairpersons of all six teams were interviewed at posttest, and in five cases the co-chair participated in these interviews as well. At pretest, 29 questionnaires were completed, at posttest 49. In addition, a focus group meeting was conducted with four participating team members, other than the chairpersons. Additionally, three team members who were not able to attend the focus group meeting were interviewed individually within two weeks after the meeting.

**Table 2:** Characteristics of the participating interprofessional primary care team meetings.

Team	Duration of meetings in minutes	Frequency of team meetings	Number of participants attending posttest observation	Disciplines

1	60	Once every two weeks	10	Case manager for dementia (1), remedial educationalist (1), district nurse (1), family physician (2), occupational therapist (1), psychologist (1), social worker (1), practice nurse (1), nurse specialist (1)
2	60	Once a month	9	Family physician (4), trainee family physician (1), practice nurse (1), physical therapist (1), district nurse (2)
3	60	Once every six weeks	12	Family physician (2), physical therapist (1), occupational therapist (1), district nurse (1), location manager (1), practice nurse (3), case manager for dementia (2), care process supervisor (1)
4	60	Once every two months	14	Family physician (1), case manager for dementia (1), practice nurse (2), physical therapist (2), occupational therapist (2), trainee occupational therapist (1), pharmacist (1), customer adviser (1), district nurse (3)
5	60	Once every two months	7	Family physician (2), practice nurse (1), physical therapist (1), doctor’s assistant (1), case manager for dementia (1), nurse (1)
6	60	Once every six weeks	8	Physical therapist (3), occupational therapist (1), district nurse (3), case manager for dementia (1)

**Table 3:** Data collection.

Team	Team meeting observations(*n* = 11)		Interviews with chairpersons and co-chairs (*n* = 6)	Number of pages transcribed	Completed questionnaires and response rate (*n* = 78)		Focus group meeting (*n* =7)

	*Pretest (n = 5)*	*Posttest (n = 6)*	*Post-test (n = 6)*		*Pretest (n = 29)*	*Post-test (n = 49)*	

**1**	X	X	Family physician	15	4 (57%)	6 (86%)	Practice nurse
**2**	X	X	Family physician (2)**	20	4 (40%)	9 (90%)	District nurse***
**3**	X	X	Family physician and practice nurse**	14	6 (67%)	8 (89%)	Care process supervisor and district nurse
**4**	X	X	Practice nurse and family physician**	20	7 (50%)	9 (64%)	Practice nurse***
**5**	X	X	Practice nurse and family physician**	11	8 (67%)	10 (83%)	Practice nurse
**6**	N/A*	X	Physical therapist and district nurse**	20	N/A*	7 (88%)	Physical therapist***

* Team 6 was set up at baseline. ** Chairperson and co-chair were interviewed together. *** Individual interview.

This description of the results starts with the evaluation of the training activities. The results are presented below for each objective for improvement: knowing each other personally, clear structure and organization, person-centredness, feedback and team reflexivity, chairing meetings and guiding team development. Lastly, we present the findings of the questionnaire regarding the programme’s potential impact.

### Participation of chairs and co-chairs in training activities

#### Level of implementation

A team instruction meeting was arranged for each team. All 12 chairpersons (chairs and co-chairs) participated actively in two training sessions, two peer feedback sessions and an on-the-job coaching session. During the training sessions, the chairpersons received instructions on, and experimented with, using the reflection framework and supporting tools as intended.

#### Participants’ experiences?

Overall, chairpersons expressed positive experiences regarding both content and form of the training course and did not feel crucial elements had been missing. They considered the variety of exercises, practice and theory in the training course to be effective. They especially regarded the theory concerning group processes (levels of communication, group development) as helpful when guiding teams. They mentioned that they had become more aware of group processes, more alert to specific behaviours of team members and better able to observe team functioning. Others perceived the training activities as a format supporting awareness of the patients’ perspective, by offering insights into theory and best practices.

*‘I think you should see the training course as a format or framework giving you some awareness of group processes and of the way this could work in an ideal situation.’* (*Family physician, chair of team 1*)

Some argued that the content of the training course should be more tailored to the specific team context (including setting, composition and organization). They mentioned that time efficiency could be improved by focusing on the efficiency-related learning objectives expressed by the team. For example, one chairperson, who was struggling with chairing a large group, suggested that more attention should have been paid to this specific theme during the training sessions.

The chairpersons especially valued the on-the-job coaching by the independent observers after the meetings, detecting blind spots and providing feedback. The chairs suggested expanding this element of the training course and would value feedback to the team during the meetings.

Participating in the training course with a colleague and co-chair was considered by the chairpersons as not only useful but also pleasant, since they were able to support each other. Chairs valued feedback from their own colleague, as well as from the participants of other teams, as being both constructive and instructive. Chairpersons suggested making more use of team activities, like refresher courses, which might inspire team members to get involved in team development.

*‘Well, I just like it that she, err, has the same information as I, so there’s always someone to fall back on, who knows how it works.* (*District Nurse, chair of team 6*)

In contrast to the request for more training activities, some chairpersons stated that participation had been time-consuming, and suggested reducing the duration of the training course. They suggested that time could be saved by shortening the theoretical part of the training course and providing additional video materials instead.

### Knowing each other personally

#### Level of implementation

On average, 10 professionals from different disciplines participated during each of the IPT meetings we observed. Observations showed that most participating teams took some time to get to know each other. There were no major differences between pretest and posttest assessments. Almost all teams conducted an introduction round when new team members joined. However, these introduction rounds were limited to presenting names and disciplines and did not comprise specific expertise, background and interests. In one of the teams, the chairperson introduced the team to the new team member, and provided the new member with background information on the composition and history of the team, and the team’s rules and agreements. Additionally, some teams developed a ‘face book’ including professional and personal details of all team members plus a photo. Compared to the observations during the pretest, the team members did not seem to have become more able to ask each other specific questions at posttest.

#### Participants’ experiences?

The interviews indicated that the chairpersons perceived getting to know each other as the most important aspect of IPT meetings. They mentioned that meeting each other physically at regular intervals helps them get to know each other personally, resulting in short communication lines. The team members also tended to contact each other more easily outside the team meetings. As an example, they perceived an increase in bilateral consultations.

*‘If you know each other, it is easier to contact each other outside the IPT meetings.’* (*Practice Nurse, Chair of team 3*)

Others mentioned that their team worked on getting to know each other by organizing yearly team excursions. Another participant perceived a closer partnership among team members of her team. However, one chairperson mentioned that her team members did not know each other that well, as the team was still in the initial phase of group development.

### Clear structure and organization

#### Level of implementation

Compared to the pretest, the meetings observed in the posttest assessment appeared to be more structured and better organized. Organizational roles were clearly divided, teams were working according to a set agenda, and some of them appointed a permanent secretary (responsible for taking notes). Chairpersons were fulfilling their role by structuring, summarizing and managing time, which was in clear contrast with most pretest meetings, where content was presented ad hoc, and mainly included announcements. At posttest, we observed less redundant talk and discussion of irrelevant matters. Meetings appeared to be better focused, resulting in clearer agreements.

Participants appeared to find it difficult to use the various tools to structure the meeting. Some teams used the toolbox form to prepare the meeting and introduce patients, others used a tailored version, while some teams did not use the tools at all. Only in one team was the brochure (including information on the different tools) placed on the table.

#### Participants’ experiences?

The chairpersons perceived improving the structure and organization of their meetings as highly relevant and felt they had improved most on this issue as result of taking part in the programme. the chairpersons described their new way of working as being more efficient.

*‘I now increasingly come out of these meetings feeling yes, that was rather efficient.* (*Family Physician, chair of team 4*)

The chairpersons stated that preparation of the meeting and agenda setting ensured that team members knew beforehand what patients and topics would be discussed, offering them the opportunity to focus their preparation. The team members also perceived improved team meeting efficiency thanks to better agenda setting and division of tasks. It was especially the agenda setting that had made the distinction between announcements and cases to be discussed in detail clearer.

*‘I think the chairpersons now work in a more structured way. They clearly distinguish between, err, when someone want to bring something up, is this an announcement or is it discussing a case, right? And, err, well if it’s just an announcement, it is quickly dismissed, and then, on we go.’* (*Care Process Supervisor, team member of team 3*)

One team member commented that the structure could be improved further by adding a time component to the agenda. This respondent also questioned whether their team should include a large number of participants, and recommended that team members should be invited selectively, based on the agenda. Furthermore, one chair reported a negative experience in that she constantly had to send reminders to the other team members to submit patient cases in time. Moreover, the chairpersons stated that it is hard to fulfil both roles, chairperson and secretary. In this respect, the suggestion to appoint a permanent and separate secretary was positively commented on by the participants.

The participants’ opinions diverged regarding the use of the tools to prepare and structure the meeting. The chairpersons valued the brochure as highly useful and feasible, but often just forgot to bring it to the meeting. Some team members perceived that the tools offered added value for the efficiency of the meetings, while others mentioned adverse effects of preparing the meeting by means of a time-consuming form. Some participants also experienced using the supporting tools as extra work, since it could not be integrated into their normal work routines. Other team members were not aware of the existence of some of the tools. Additionally, the chairpersons mentioned the negative impact of bureaucracy on their work routines, and warned against an overload of forms and protocols.

### Person-centredness

#### Level of implementation

Compared to the findings at pretest, some practices appeared to have become more focused on the patient’s perspective, by frequently asking: *‘What do we know about the patient’s needs?’* We also observed that some teams paid special attention to the privacy of the patients discussed. They mentioned that the patients should be informed beforehand and asked for permission to present their cases during the IPT meeting. They also explicitly incorporated agreements on privacy in their working protocols. In none of the meetings did a patient or a representative take part. The tool to enhance person-centredness (the six-step plan) was only occasionally used as intended. In most of the cases discussed, the goals of the patient (step 2) and the analysis of the situation, including aspects of self-management (step 3) were not explicitly mentioned. Only in exceptional cases did the teams use the placemat presenting a description of the six-step plan. For some of the cases discussed, the team suggested to present the options to the patient in order to enhance shared decision making. However, in other cases the team decided upon actions without the patient’s direct approval.

#### Participants’ experiences?

After implementation, participants reported to have become more aware of the patient’s perspective and to have developed increased awareness of the rationale of their contribution. Some stated that they had become more aware of privacy issues regarding sharing patient information.

*‘I now probably, perhaps still not enough, but certainly more than before, think more about what the patients themselves want. What their wishes are, or their goals, and I notice that we still often talk from a medical perspective, what we think is good for people, but the alertness and the fact that people have preferences, that I now think about that more often. And that feels very good.’* (*Family Physician, chair of team 5*)

However, chairpersons experienced a lack of patient and family involvement before, during and after team meetings. They expressed that, for instance, in the case of care avoiders, it is often difficult to explore a patient’s goals and get their permission to present the patient during an IPT meeting. Participants reported that the professional perspective often dominates especially in those cases. Regarding the tools, chairpersons mentioned that the form used to present patient cases was easy to use, and indicated that using the form made team members more aware of their contributions. However, team members often wanted to present and discuss patients ad hoc, leaving less time to fill in the form. Since using the form, some members had detected a decrease in the number of patients being presented during their meetings. In order to avoid the form becoming a threshold to presenting patients, some teams adjusted the form and applied it less strictly.

*‘No, we didn’t use the actual form, but used a number of items from it, which we regard as essential for us.’* (*Family Physician, chair of team 1*)

Some of the team members experienced the six-step plan as useful and as offering added value. Others regarded the six-step plan as too extensive and they mentioned lacking the time to go through all six steps. Some mentioned that they did not follow all steps as intended and explained that they still had to get used to the new way of working.

*‘The disadvantage is that it takes more time. It has, has to be filled in beforehand, you need to think about it beforehand, right? You can’t just come to an IPT meeting unprepared.’* (*Practice Nurse, team member of team 4*)

*‘I try to think about it, to focus on the client, but sometimes it, it just disappears, as you allow yourself to get carried away by the content of the questions discussed by the care providers.’* (*District Nurse, chair of team 6*)

### Feedback and team reflexivity

#### Level of implementation

Three of the six teams observed at posttest reflected on their functioning. One of the teams put reflection on the agenda. All team members were given the opportunity to provide input and express their thoughts. Regarding team climate, we observed a pleasant atmosphere and informal working environment in all teams, allowing reflection and enabling team members to give each other feedback. Chairpersons rarely attempted to ask in-depth questions. Consequently, reflection remained superficial, with personal opinions and experiences not explored in depth.

#### Participants’ experiences?

Most chairpersons acknowledged the added value of team reflection in relation to team development. One team member mentioned a positive experience regarding reflection on the application of the form.

*‘Yeah, that’s the main thing. Not just thinking this is it, but to keep searching to see, well, this is where we stand, what can we improve?’* (*Family Physician, chair of team 4*)

However, most chairpersons mentioned that reflection did not occur often enough, which was confirmed by other team members. One team member stated that initiating reflection is the chairpersons’ job, and that he did not feel responsible for initiating reflection and improving team functioning.

*‘A sense of responsibility also has to do with a sense of binding, of engagement. You see, we just sit there once a month. And if you miss a meeting, it’s once in two months.’* (*District Nurse, team member of team 2*)

One chairperson stated that reflection was intended to be part of the any other business round at the end of the meeting, but in practice this round was used for other content-related matters. Others perceived reflection on team functioning as difficult due to the continuously changing team composition. Some chairpersons perceived added value of reflection on problem situations. Moreover, the chairpersons reported that if reflection was needed, it should be initiated spontaneously during the meeting itself and not be scheduled at fixed reflection moments. Lastly, the participants perceived an open and safe team climate, with plenty of room for reflection.

### Chairing meetings and guiding team development

#### Level of implementation

Our observations showed that most chairs at posttest were more explicitly structuring the meetings and guiding their team. Compared to pretest, chairs summarized and paraphrased more often at posttest. We also observed a growth in leadership skills. Some chairs were noticeably inspiring and involving other team members to take part in the discussion, by recognizing and appreciating their contributions. Most were contributing to a positive atmosphere and healthy working environment, by acting as a host.

#### Participants’ experiences?

Most chairpersons enjoyed developing their skills to guide the team. They felt that attending the programme had enabled them to structure the meetings and guide team development. Some mentioned that their role had changed into ‘the team’s driving force’. In addition, the chairpersons mentioned that they had become more aware of group processes and specific behaviours, and better able to observe processes. Conversely, team members did not perceive huge changes in the role and position of their chairpersons. Some reported to have noticed that their team’s chairperson was still experimenting. Other team members mentioned to have become more confident, empowered and active and more willing to express their opinions, since they had more practical tools.

*‘Taking part in this programme gave us a boost to continue conducting IPT meetings.’* (*Family Physician, chair of team 3*)

Some team members mentioned the positive effect of the natural interplay between the chairperson and the co-chair. However, chairs also experienced that chairing meetings and guiding the team takes a lot of time, and is not always an easy task. A team member remarked that in their team two chairpersons were alternating with each other, and experienced this as rather confusing.

*“I wasn’t really aware that chairing the meetings would be such a different task for me. I just thought, well, I’ll just do it. Well, and it wasn’t like that. You don’t just do it without effort.’* (*Physical therapist, chair of team 6*)

None of the participating chairs reported finding it difficult to deal with irritations or annoying situations, although one team member perceived the dominance of one of the family physicians (not the chairperson) as a barrier to collaboration. One chair mentioned struggling with the large team size. Another chair mentioned that as a chairperson you constantly have to adjust to who is attending the meeting, questioning whether the team could be really considered a team given its continuously changing composition.

‘Actually, I wonder whether you could call this a team at all, since there’s a constant change of the people attending. Although they represent the disciplines we expect at that moment, it’s never the same team. There are always different people, which means different perspectives.’(Practice Nurse, chair of team 2)

### Programme’s potential impact on team climate, efficiency and person-centredness

Table 3 offers an overview of the response rates and numbers of questionnaires collected, for each team. The main results for each section of the questionnaire are presented in Table 4. An overview of the scores on the individual questions per practice can be found in Supplementary file 4. Table 4 shows that pretest scores differed between the teams. Table 4 shows a small increase between pretest and posttest for all domains. The largest increase was found in the domain of ‘Task Style’, relating to team climate and including questions on team reflexivity. Differences between teams were small. However, as becomes clear from Table 4, team 1 had the largest number of highest scores at both pretest and posttest. Team 1 and team 5 showed a decrease on some domains, which may be explained by the fact that these teams had a longstanding, structured practice regarding conducting IPT meetings. Participation in the programme seemed to have induced reflection on their routines. Team 6 had the lowest scores on most parts of the questionnaire. At the level of individual items, the highest absolute increase, 0.71 (pretest = 5.07 and posttest = 5.78), was found for question 45: *‘The way we work during team meetings can be considered efficient’*.

**Table 4:** Mean scores of the questionnaire per team.

Mean scores of the 6 participating teams *Sum scores per domain*	No. of Questions	Range	Team 1 (10 members)		Team 2 (9 members)		Team 3 (12 members)		Team 4 (14 members)		Team 5 (7 members)		Team 6 (8 members)	

Measurement			Pre N = 4	Post N = 9	Pre N = 6	Post N = 8	Pre N = 8	Post N = 10	Pre N = 7	Post N = 9	Pre N = 4	Post N = 6	Pre N = 0	Post N = 7

Communication and innovation	20	1–5	4.18	3.94	3.86	3.91	3.45	3.88	3.62	3.95	3.98	3.87	–	3.69
Objectives	10	1–5	4.20	4.55	4.07	4.23	3.57	4.01	3.68	4.23	4.34	4.13	–	4.27
Task style	8	1–5	4.10	4.16	3.28	3.79	3.08	4.21	3.14	3.87	4.14	3.88	–	3.76
Person-centredness	6	1–7	5.58	5.57	5.00	5.38	5.12	5.18	4.98	5.33	5.63	5.17	–	5.10
Team efficiency	3	1–7	6.33	6.26	5.06	5.50	4.81	5.37	5.05	5.74	5.75	5.44	–	5.48
Team functioning	1	Grade1–10	9	8.39	7.33	7.63	6.71	7.25	7.07	7.67	7.75	7.67	–	7.43

## Discussion

This process evaluation has yielded insights into the suitability and potential impact of a programme intended to improve the functioning of interprofessional team (IPT) meetings in primary care, focusing on five main objectives for improvement: (1) knowing each other personally, (2) clear structure and organization, (3) person-centredness, (4) feedback and team reflexivity, and (5) chairing meetings and guiding team development. The findings contribute to a good understanding of the programme’s usefulness and offer suggestions to optimize the programme.

In general, our findings showed that the programme was suitable and well appreciated. The programme resulted in improved structure and organization of the meetings. Most progress was made on efficiency, by improved preparation, agenda setting, time use, and increased focus. Moreover, the programme resulted in greater awareness of the value of the patient perspective and the team processes. However, apart from increased awareness, no great impact was seen in the observations on person-centredness in terms of explicitly exploring patient goals. There seems to be a discrepancy between what professionals do and what they think they do regarding person-centredness. These findings appear similar to those of our previous study during the needs assessment, which also showed discrepancies between observations and interviews regarding efficiency and person-centredness [6]. In some teams, the number of patients discussed during the meetings had decreased. Participants explained that the new approach challenges the team members to carefully consider the patient’s goals and their level of complexity beforehand, resulting in more consciousness regarding patients discussed. Others stated that the decrease was caused by the threshold formed by the obligation to prepare the meeting according to a structured form, and the extra time this takes. The tools we provided to enhance person-centredness may be too complex or time-consuming for some team members. Simplified tools, tailored to the needs of the team, seem desirable. It is likely that increasing person-centredness and integrating new ways of working into existing work processes requires time and behavioural change among the participating professionals.

Within our programme, the chairpersons were positioned as key figures, who were intended to operate as role models and change agents, enabling the team to improve its own functioning. The chairpersons were trained and facilitated to organize and structure meetings, and enhance person-centredness by showing effective leadership and using the tools. They were trained to adapt their style of leadership (directive, convincing, participatory, or delegating) to the specific team context and the phase of the group development process, which is known as situational leadership [26]. Different leadership styles were observed, however, most chairpersons actually adopted a more directive style of leadership. Further, it is known that seniority of grade is associated with a higher quality of leadership behaviour [27]. This view could be reasonable, since the findings of our questionnaire show high scores for team 1, which was chaired by two physicians.

Although programme components and materials were perceived as valuable, some chairs experienced difficulties in implementing their new role, and did not feel able to guide their team through development. Chairpersons of the less stable teams reported being confronted with a continuously changing team composition and frequent absence of core team members. Given this changing team composition, it appears questionable whether all interprofessional primary care teams can be considered teams, or whether it would be better to regard some of them as networks [28]. In this respect, Lingard talks about distributed teams composed of members from various organizations who may not know each other and have restricted opportunities to develop shared values compared to acute care teams [29].

The dynamic composition of some teams also hampered their ability to reflect upon and adjust team functioning. However, there are more reasons why team reflection was not conducted as intended. Some chairpersons experienced problems initiating reflection moments, or did not perceive any added value. As a possible strategy to enhance reflexivity, participants mentioned the value of an external facilitator who, compared to the chairperson, would as an outsider be better able to identify and comment on problem points. In this respect, participants also suggested to expand the on-the-job learning activities in which the entire team is coached. Other studies have also shown that supporting change by means of reflection is more effective when IPT meetings are mediated by a skilled facilitator, e.g. a researcher/consultant who is familiar with the specific team context [30,31]. Transformational leadership, characterized by a leader who inspires and motivates other team members regarding their vision, goals and action plans, is also positively related to team reflection [32,33]. Adopting principles of this approach to leadership in our programme can therefore be a suitable strategy to stimulate team reflexivity and adapting to a rapidly changing environment [32,33]. Moreover, since people tend to follow good examples, the chairs/leaders in our programme could act as role models and change agents [34]. However, further research on interprofessional team leadership is needed to increase knowledge on the degree to which adequate leadership leads to better patient related outcomes [35]. In their review, Smith and colleagues conclude that effective interprofessional team leadership requires a unique mix of competences supporting innovation and improvement [35].

Lastly, interprofessional primary care teams differ in context, in terms of setting, composition, organization, and phase of development. Our findings did indeed appear to vary under the influence of the professional and organizational contexts of the teams. Harris and colleagues also found differences in the impact of programmes to enhance interprofessional teamwork, due to contextual differences [13]. Even more than anticipated, the findings of our evaluation indicate that both content and form of the programme should be flexible in use, adapted to the team context, and tailored to its specific needs. Other studies have found similar results [14,15,36]. Conducting an intake procedure at the start of the programme, including a thorough assessment (including needs assessment and observation), could offer insights into these specific team contexts and needs. Outcomes of this intake procedure can then be used to shape and tailor the content and form of the programme.

### Strengths and limitations

We used a systematic approach to design and conduct our process evaluation, using the Medical Research Council (MRC) Guide [17]. Our use of different forms of data collection (observations, interviews, focus groups and questionnaires) helped to clarify the complex pathways and contextual factors. Some of the interviews with chairpersons included both chairperson and co-chair. Interviewing them together may have led to less forthright answers. However, the interviewees appeared to elaborate on each other’s answers, resulting in additional viewpoints. Our findings show some discrepancies between the experiences of chairpersons and those of the other team members. This can be explained by the fact that the chairpersons had all practiced with the various tools we offered, while some of the team members were not even aware of the existence of some of the tools.

The fact that the input for this process evaluation was a systematic needs assessment in which the programme had been developed in close collaboration with all stakeholders (using an action research approach), can be regarded as strength of our study. Nevertheless, this does not remove the need for a thorough process evaluation when a programme is implemented in daily practice.

During the needs assessment observation and interview guides had already been evaluated, which can also be regarded as a strength of our study [6,7,8]. The observations were conducted by two independent researchers, as recommended by Moore et al. [17].

The researcher (JvD) who observed the meetings and interviewed participants also functioned as trainer/coach. Ideally, according to the MRC guide, researchers should not engage in continuous quality improvement activities, because this may compromise the external validity of the evaluation. However, in our case the researcher acted as a passive observer, and did not intervene or feed findings back during or after the team meeting, minimizing the effects of this possible limitation [37].

Although only a limited number of teams and members were included and were selected by convenience sampling, the teams varied considerably regarding composition and organization which reflects the situation in primary care. The average score for team functioning at baseline was relatively high for some of the teams, which may imply that we included mainly well-functioning teams. However, the observations at baseline showed that all teams required some improvement. Given the average frequency of meetings and the time needed to change behaviour, the follow-up of the teams can be regarded as relatively short.

Moreover, given the scope of this study, findings were derived in the context of the Dutch primary care setting. We are not able to conclude on generalizability to other health care settings or countries, however since most participating primary care teams differ from each other in composition and approach, and are influenced by various contextual factors, we expect that the findings are transferable to other primary care settings. On a more abstract, theme level, findings appear to be transferable to other settings, such as community care.

To end, due to the process evaluation character of this study, the differences between the teams, and the low number of respondents, the quantitative data were only analysed descriptively. Given this design, we did not evaluate the effect of the programme, but offered some early insights into its potential impact.

## Conclusion

The programme appears suitable for improving interprofessional team functioning. Overall, the programme improves both the structure and organization of interprofessional primary care team meetings. The programme also contributed to increased awareness of person-centredness and group processes among the participants. It is likely that increasing team reflexivity and person-centredness, and integrating new ways of working in existing work processes, will require time among the participating professionals. Furthermore, to be more effective, the programme should be even more context-specific and flexible in use than anticipated, and asks for an intake procedure at the start, and support and coaching by an external facilitator at the workplace. Given the perceived added value of the peer feedback and on-the-job coaching of the entire team, it appears worthwhile to improve these components of the programme.

## Additional Files

The additional files for this article can be found as follows:

10.5334/ijic.4179.s1 Supplementary file 1. Questionnaire.

10.5334/ijic.4179.s2 Supplementary file 2. Observation guide.

10.5334/ijic.4179.s3 Supplementary file 3. Interview guide.
