# Supplementary material for: Suitability of a Programme for Improving Interprofessional Primary Care Team Meetings
Source: Int J Integr Care. 2018 Dec 13;18(4):12. doi: 10.5334/ijic.4179 (PMC6293205; doi:10.5334/ijic.4179)
Supplement: Supplementary file 1. — Questionnaire. [file ijic-18-4-4179-s1.pdf]

	Total group (N=78)		Team 1 (N=13)		Team 2 (N=14)		Team 3 (N=18)		Team 4 (N=16)		Team 5 (N=10)		Team 6 (N=7)	
	Mean		Mean		Mean		Mean		Mean		Mean		Mean	
	T0 (N=29)	T1 (N=49)	T0 (N=4)	T1 (N=9)	T0 (N=6)	T1 (N=8)	T0 (N=8)	T1 (N=10)	T0 (N=7)	T1 (N=9)	T0 (N=4)	T1 (N=6)	T0 (N=0)	T1 (N=7)
**Part 1.1 Communication and innovation (DTCI) (range 1-5)**														
1. We share information generally in the team rather than keeping it to ourselves.	4.38	4.31	4.75	4.67	4.67	4.25	4.00	4.20	4.43	4.22	4.25	4.33		4.14
2. Assistance in developing new ideas is readily available.	3.76	3.88	4.50	4.00	3.50	3.88	3.75	3.80	3.43	3.89	4.00	3.83		3.86
3. We all influence each other.	3.93	3.96	4.25	3.89	3.67	4.00	3.63	3.80	4.29	4.11	4.00	4.00		4.00
4. We keep in regular contact with each other.	4.07	4.06	4.50	3.78	4.33	4.25	3.88	4.20	3.86	4.11	4.00	3.83		4.14
5. In this team we take the time needed to develop new ideas.	3.10	3.71	3.50	3.67	3.17	3.13	2.63	4.00	3.14	3.89	3.50	3.50		4.00
6. People feel understood and accepted by each other.	4.07	4.22	4.75	4.67	4.00	4.00	3.75	4.30	4.14	4.00	4.00	4.17		4.14
7. Everyone's view is listened to, even if it is in a minority.	4.10	4.22	4.75	4.67	4.17	4.00	3.88	4.20	4.00	4.11	4.00	4.33		4.00
8. The team is open and responsive to change.	4.03	4.14	4.50	4.33	4.00	4.00	3.75	4.20	4.00	4.11	4.25	4.17		4.00
9. People in the team co-operate in order to help develop and apply new ideas.	3.55	3.94	4.00	3.78	3.67	4.25	3.00	4.20	3.71	4.11	3.75	3.50		3.57
10. We have a 'we are in it together' attitude.	4.14	4.29	4.50	4.67	4.33	4.38	3.75	4.10	4.00	4.22	4.50	4.50		3.86
11. We interact frequently.	3.21	3.22	3.75	3.00	3.50	3.50	2.75	3.30	2.86	3.56	3.75	3.00		2.86
12. People keep each other informed about work-related issues in the team	4.00	3.82	4.25	3.78	4.17	4.13	4.00	3.80	3.57	4.00	4.25	4.00		3.14
13. Members of the team provide and share resources to help in the application of new ideas	3.45	3.67	3.50	3.89	3.83	3.50	3.13	3.80	3.29	3.89	3.75	3.67		3.14
14. There is a lot of give	3.55	3.61	4.50	3.67	3.50	3.88	3.38	3.60	3.14	3.78	3.75	3.50		3.14

and take														
15. We keep in touch with each other as a team	3.93	3.90	4.00	3.89	4.17	4.13	3.75	3.90	3.86	3.78	4.00	3.83		3.86
16. People in this team are always searching for fresh, new ways of looking at problems	3.52	3.82	4.00	3.89	3.67	3.75	3.13	3.60	3.29	4.11	4.00	3.83		3.71
17. There are real attempts to share information throughout the team.	4.14	4.16	4.50	4.33	4.00	4.00	4.00	4.10	4.14	4.33	4.25	4.17		4.00
18. This team is always moving towards the development of new answers	3.52	3.92	4.25	3.89	3.50	4.00	3.13	3.90	3.43	4.00	3.75	4.00		3.71
19. Team members provide practical support for new ideas and their application.	3.79	3.98	4.25	3.89	4.17	3.88	3.38	4.00	3.71	4.11	3.75	4.17		3.86
20. Members of the team meet frequently to talk both formally and informally.	2.83	2.78	2.75	2.56	3.33	3.38	2.38	2.60	2.14	2.67	4.25	3.00		2.57
	**Total group** (N=78)		**Team 1** (N=13)		**Team 2** (N=14)		**Team 3** (N=18)		**Team 4** (N=16)		**Team 5** (N=10)		**Team 6** (N=7)	
	Mean		Mean		Mean		Mean		Mean		Mean		Mean	
	T0 (N=29)	T1 (N=49)	T0 (N=4)	T1 (N=9)	T0 (N=6)	T1 (N=8)	T0 (N=8)	T1 (N=10)	T0 (N=7)	T1 (N=9)	T0 (N=4)	T1 (N=6)	T0 (N=0)	T1 (N=7)
**Part 1.2 Objectices (DTCI) (range 1-5)**														
21. How clear are you about what your team objectives are?	3.93	4.35	4.50	4.78	4.20	4.38	3.50	3.90	3.57	4.33	4.50	4.17		4.57
22. To what extent do you think they are useful and appropriate objectives?	3.96	4.37	4.50	4.78	4.20	4.50	3.75	3.90	3.67	4.56	4.00	4.00		4.43
23. How far are you in agreement with these objectives?	4.04	4.47	4.50	4.89	4.20	4.38	3.63	4.10	3.83	4.56	4.50	4.33		4.57
24. To what extent do you think other team members agree with these objectives?	3.96	4.20	4.00	4.56	4.20	4.38	3.57	3.80	3.83	4.11	4.50	4.33		4.14

25. To what extent do you think your team's objectives are clearly understood by other members of the team?	3,93	4.14	4.25	4.56	4.00	4.25	3.63	3.60	3.67	4.33	4.50	4.00		4.14
26. To what extent do you think your team's objectives can actually be achieved?	3.85	3.94	4.00	4.33	4.00	3.75	3.71	4.20	3.86	3.56	3.75	3.83		3.86
27. How worthwhile do you think these objectives are to you?	4.04	4.31	4.25	4.56	4.00	4.38	3.88	4.00	3.83	4.44	4.50	4.00		4.43
28. How worthwhile do you think these objectives are to the organization?	4.04	4.35	4.25	4.44	4.20	4.50	3.88	4.20	3.67	4.33	4.50	4.17		4.43
29. How worthwhile do you think these objectives are to the wider society?	4.04	4.31	4.25	4.56	4.20	4.38	3.88	4.10	3.67	4.33	4.50	4.20		4.29
30. To what extent do you think these objectives are realistic and can be attained?	3,85	4.04	4.00	4.56	4.00	3.50	3.63	4.00	3.67	4.11	4.25	4.00		4.00
31. To what extent do you think members of your team are committed to these objectives?	3.67	4.08	3.75	4.00	3.60	4.13	3.71	4.30	3.29	3.89	4.25	4.00		4.14
	**Total group**		**Team 1**		**Team 2**		**Team 3**		**Team 4**		**Team 5**		**Team 6**	

	(N=78)		(N=13)		(N=14)		(N=18)		(N=16)		(N=10)		(N=7)	
	Mean		Mean		Mean		Mean		Mean		Mean		Mean	
	T0 (N=29)	T1 (N=49)	T0 (N=4)	T1 (N=9)	T0 (N=6)	T1 (N=8)	T0 (N=8)	T1 (N=10)	T0 (N=7)	T1 (N=9)	T0 (N=4)	T1 (N=6)	T0 (N=0)	T1 (N=7)
**Part 1.3 Task style (DTCI) (range 1-5)**														
32. Do your team colleagues provide useful ideas and practical help to enable you to do the job to the best of your ability?	4.18	4.37	4.50	4.44	4.00	4.38	4.00	4.50	4.00	4.33	4.75	4.33		4.14
33. Do you and your colleagues monitor each other so as to maintain a higher standard of work?	3.77	4.06	4.25	4.33	3.80	3.88	3.29	4.30	3.50	3.78	4.50	4.17		3.86
34. Are team members prepared to question the basis of what the team is doing?	3,44	4.19	4.00	4.38	3.40	4.00	3.00	4.60	3.57	3.78	3.50	4.00		4.29
35. Does the team critically appraise potential weaknesses in what it is doing in order to achieve the best possible outcome?	2.44	3.00	2.75	3.33	2.33	2.50	2.33	3.30	2.00	2.89	3.25	3.00		2.83
36. Do members of the team build on each other's ideas in order to achieve the best possible outcome?	3.67	4.12	4.50	4.33	3.60	4.00	3.20	4.30	3.00	4.11	4.50	4.00		3.86
37. Is there a real concern among team members that the team should achieve the highest standards of performance?	4.00	4.24	4.50	4.44	3.83	4.50	3.67	4.50	3.86	4.22	4.50	4.00		3.57
38. Does the team have clear criteria which members try to meet in order to achieve excellence as a team?	2.81	3.85	4.25	4.00	2.33	3.25	2.33	4.22	2.00	4.00	4.00	3.67		3.86
	**Total group**		**Team 1**		**Team 2**		**Team 3**		**Team 4**		**Team 5**		**Team 6**	

	(N=78)		(N=13)		(N=14)		(N=18)		(N=16)		(N=10)		(N=7)	
	Mean		Mean		Mean		Mean		Mean		Mean		Mean	
	T0 (N=29)	T1 (N=49)	T0 (N=4)	T1 (N=9)	T0 (N=6)	T1 (N=8)	T0 (N=8)	T1 (N=10)	T0 (N=7)	T1 (N=9)	T0 (N=4)	T1 (N=6)	T0 (N=0)	T1 (N=7)
**Part 2 (range 1-7)**														
39. Team members encourage patients/clients to be active participants in care decisions.	5.21	5.41	6.00	5.78	4.17	5.25	5.14	5.70	5.29	5.44	6.00	4.50		5.43
40. Team members meet face-to-face with patients/clients cared for by the team.	6.25	6.04	6.00	6.44	6.83	6.38	6.00	6.10	6.14	5.56	6.25	6.17		5.57
41. Information relevant to health care planning is shared with the patient/client.	5.39	5.47	6.25	5.33	5.33	5.75	5.43	5.30	4.86	5.22	5.50	5.67		5.71
42. The patient/client is considered a member of their health care team.	3.50	4.08	3.50	4.00	2.83	3.75	3.29	4.30	3.71	4.56	4.50	3.83		3.86
43. The patient's/client's family and supports are included in care planning, at the patient's request.	5,61	5.69	6.25	5.89	5.00	5.75	5.71	5.60	5.29	5.33	6.25	5.83		5.86
44. Members of our team have a good understanding of patient/client care plans and treatment goals.	5.21	5.10	5.50	6.00	5.83	5.38	5.14	4.10	4.57	5.89	5.25	5.00		4.14
45. Our way of working during team meetings can be considered efficient.	5.07	5.78	6.25	6.33	4.50	5.50	4.57	5.40	5.00	6.11	5.75	5.33		5.86
46. Our team meetings lead to good outcomes.	5.46	5.86	6.50	6.22	5.33	5.88	4.86	5.50	5.29	6.00	6.00	5.83		5.71
47. The shared care plans link with the patients' personal wishes and preferences.	5.29	5.31	6.25	6.22	5.33	5.13	5.00	5.20	4.86	5.11	5.50	5.17		4.86
48. Score for overall team functioning (range 1-10)	7.411	7.673	9.000	8.389	7.333	7.625	6.714	7.250	7.071	7.667	7.750	7.667		7.429

	Total group (N=78)		Team 1 (N=13)		Team 2 (N=14)		Team 3 (N=18)		Team 4 (N=16)		Team 5 (N=10)		Team 6 (N=7)	
	T0 (N=29)	T1 (N=49)	T0 (N=4)	T1 (N=9)	T0 (N=6)	T1 (N=8)	T0 (N=8)	T1 (N=10)	T0 (N=7)	T1 (N=9)	T0 (N=4)	T1 (N=6)	T0 (N=0)	T1 (N=7)
**Total mean scores per section**														
Mean scores Part 1.1: Q1-20 (range 1-5)	3.75	3.88	4.18	3.94	3.86	3.91	3.45	3.88	3.62	3.95	3.98	3.87		3.69
Mean scores Part 1.2: Q21-31 (range 1-5)	3.90	4.24	4.20	4.55	4.07	4.23	3.57	4.01	3.68	4.23	4.34	4.13		4.27
Mean scores Part 1.3: Q 32-38 (range 1-5)	3.48	3.96	4.10	4.16	3.28	3.79	3.08	4.21	3.14	3.87	4.14	3.88		3.76
Mean scores Part 2: Q39-47 (range 1-7)	5.22	5.42	5.83	5.80	5.01	5.42	5.01	5.24	5.00	5.47	5.66	5.26		5.22
Mean score team functioning: Q48 (range 1-10)	7.41	7.67	9.00	8.39	7.33	7.63	6.71	7.25	7.07	7.67	7.75	7.67		7.43

T0 = prestest
T1 = posttest
