# Supplementary material for: Suitability of a Programme for Improving Interprofessional Primary Care Team Meetings
Source: Int J Integr Care. 2018 Dec 13;18(4):12. doi: 10.5334/ijic.4179 (PMC6293205; doi:10.5334/ijic.4179)
Supplement: Supplementary file 2. — Observation guide. [file ijic-18-4-4179-s2.pdf]

Supplementary file 2. Observation guide

<table>
<tr><td>Observation list

Overall</td><td>Is the programme (core component / tool) being applied, and if so, which parts?
How (and to what extent) are the different components and tools of the programme being applied?</td><td>Manageability and feasibility
What do you notice about the feasibility of the programme?
Bottlenecks
What are the evident bottlenecks?
Contextual factors
Which visible contextual factors are influencing the application of the programme?
Added value
What can you say about the added value provided by the programme?</td></tr>
<tr><td>Knowing each other</td><td>○ Team members know each other (No one has to be introduced, no new faces)</td><td></td></tr>
<tr><td>Rules for the meeting</td><td>○ Everyone visibly adheres to the agreements and rules for the meeting
○ Team members stick to the organizational agreements</td><td></td></tr>
<tr><td>Roles</td><td>○ <u>Chairperson:</u>
○ Has prepared the meeting
○ Structures the meeting
○ Ensures adherence to the rules of the meeting
○ Follows the six-step plan
○ Summarizes
○ Keeps track of time
○ Guides reflection
○ Points out tensions
○ Clearly announces closing the meeting and plans next meeting

○ <u>Secretary:</u>
○ Takes care of the reporting (preparing minutes)
○ Checks regularly whether minutes correspond to what has been agreed on during discussion
○ Where appropriate, refers to previous minutes</td><td></td></tr>
<tr><td>Inter-professional meeting structure</td><td>○ <u>Preparation</u>
○ Clear agenda
○ Information about patients is provided before the meeting

○ <u>Meeting</u>
○ Opening
○ Discussion of each patient
○ Closing

○ <u>Aftercare</u>
○ Reporting
○ Agreements regarding feedback to patient
○ Agenda and agreements for next meeting</td><td></td></tr>
<tr><td>Interaction and team climate</td><td>○ <u>Team members give each other feedback</u>
○ On content
○ On procedure
○ On interaction

Any comments about the team climate and atmosphere?
Are there any visible irritations?
Any comments about the way the team talks about patients?</td><td></td></tr>
<tr><td>Reflection tool</td><td><u>At which level does the team reflect?</u>

○ Is there any input from participants?
○ Are specific learning objectives defined?</td><td></td></tr>
</table>

	○ Does the team refer back to previous agreements and learning objectives?	

**Observation list**<br><br>**Per patient**	**Is the programme (core component / tool) being applied, and if so, what parts?**<br>*How (and to what extent) are the different parts and tools of the programme applied?*	**Manageability and feasibility**<br>*What can you say about the feasibility of the programme?*<br>**Bottlenecks**<br>*What are the evident bottlenecks?*<br>**Contextual factors**<br>*Which visible contextual factors are influencing the application of the programme?*<br>**Added value**<br>*What can you say about the added value provided by the programme?*
Roles	**Presenter:**<br>○ Gives a clear description of the patient's background and situation<br>○ Presents the patient's personal goals and wishes<br>○ Presents an explicit question for the team<br>○ Follows the six-step plan<br><br>**Participants:**<br>○ Active participation<br>○ Clearly prepared (do not ask questions that are redundant)	
Preparation	○ Presenter has filled in the form correctly and completely (there are no questions or uncertainties)<br>○ The form has been submitted on time and according to the agreed rules	
Six-step plan	○ Description of the patient's situation<br>○ Patient's goals and motivation<br>○ Analysis<br>○ Formulating concrete proposals for action<br>○ Formulating concrete care agreements<br>○ Evaluation	
