# Supplementary material for: Suitability of a Programme for Improving Interprofessional Primary Care Team Meetings
Source: Int J Integr Care. 2018 Dec 13;18(4):12. doi: 10.5334/ijic.4179 (PMC6293205; doi:10.5334/ijic.4179)
Supplement: Supplementary file 3. — Interview guide. [file ijic-18-4-4179-s3.pdf]

Supplementary file 3. Interview guide

- Introduction
    - Introduction of the interviewer
    - Consent for audio recording
    - Explanation of participants' rights
    - Aim of the interview: The aim is to explore personal experiences (both barriers and facilitators) regarding usability and added value of the programme
    - Procedure, duration etc.
    - Emphasizing that all information is relevant

Introduction:

In the past year, your practice took part in a programme to improve interprofessional team functioning. You as the chairperson attended various training activities relating to structuring and organizing meetings and enhancing patient-centredness. Activities comprised two training sessions, peer feedback sessions and on-the-job coaching. During these activities you learned about the use of various supporting tools and then experimented with them in practice.

Subquestions:

- *Can you give an example?*
- *Can you explain in detail what you mean by…?*
- *Why do you perceive ...as a relevant factor/barrier/facilitator?*

Main questions:

Questions concern perceived use, handling, added value and corresponding facilitators and barriers to the programme.

1. *How did you feel about applying the lessons learned during the training activities in practice? How did you feel about applying the new methodology and supporting tools?*
    a. Why did you use (or not use) the components of the programme/ tools?
    b. What about their usability?
    c. What barriers did you encounter in applying the new methodology and tools?
    d. What is the added value of the different components of the programme / tools?
2. *What did the programme and new approach bring to the team?*
    a. Could you explain this? Why is that an added value?
3. *How satisfied are you with the new approach / methodology?*
    a. Why? Could you explain this? Could you give an example?
4. *How would you describe your role in the team? How did this role develop in the recent period?*
    a. To what extent did your role in the team change compared to before you took part in the programme?
    b. If applicable, could you tell me something about your personal growth. What changed? What did you learn? What would you like to learn?
    c. To what extent did the training and coaching facilitate you in this development?
    d. What supported or hampered you in acquiring the knowledge?
    e. In your opinion, what aspects were missing from the training and coaching?

    f. Could you mention components that we have to keep in the training? And could you mention aspects that could be removed?

5. *What did the programme bring you as a person?*
    a. Could you explain this?
    b. What did you learn?
    c. For what skills/competences did you develop more confidence?
    d. What new insights did the programme offer you?
    e. What are you still struggling with?
6. Is there something else you want to share regarding the programme and the various training activities?

<u>Summary:</u>

Interviewer gives a summary.

- *Did I forget any relevant information in this summary?*
